# An Attempt to Correct a Few Errors

**Published:** 1912-12-15

**Authors:** Henry W. Morgan

**Affiliations:** Nashville, Tenn.


### AN ATTEMPT TO CORRECT A FEW ERRORS.

BY HENRY W. MORGAN, M.D., D.D.S., NASHVILLE, TENN.

Read before the Tennessee Dental Association, June 7, 1912.

There appeared in the April number of *The Dental Cosmos*, two papers—one "A Reply to Dr. W. R. Black" entitled "Excavation for Prevention" and another by a learned and distinguished Philadelphian under the caption "Is Operative Dentistry Degenerating."

These papers are an attempt to answer a rather vigorous "Review" read at the National Dental Association in July last, which appeared in the January *Cosmos*, and more for the purpose of establishing the standing and professional character of the "Causus Belli," than an attack upon the principle of "Extension for Prevention."

It must not be supposed that two of Pennsylvania's leading lights would do an injustice, or for the sake of argument, or advantage, draw conclusion or make statements without a full knowledge of what has been written and is advocated in support of the scientific research and clinical observations that uphold the teachings concerning so important a factor in our work of combating and warding off recurrent caries, therby contributing to the life of the teeth and efficiency of fillings.

A note addressed to each of these gentlemen, asking if they possessed copies of Black's Operative Dentistry met with a refusal to say whether they did or did not.

It is not with any intention to provoke controversy that this paper is presented, but for the purpose of contrasting some of the statements made with what the advocate and originator of the disputed principles has published.

The writer of the second paper is of the opinion that geography has something to do with the progress the profession is making these latter days. Yes, it has! The mists that overhang the Allegheny's have obscured the vision of some of our friends and their scientific acumen is not at all clear. Nothing seems to be good that does not come from the East where the first rays of light blessed the birth of the profession. Yes, there is where we went for our training thirty years ago. In those days it was an unselfish search for truth,—"They painted, for the Master the things as they saw them."

A careful study of the work of Dr. Black reveals that it is based on an accurate knowledge of anatomy, histology and pathology, learned in the laboratory and at the chair, and the testimony of all who have weighed well his work, is that his conclusions are logically and truthfully portrayed. Leave behind all preconceived theories, all personal beliefs and hobbies, and you will be forced to the opinion that his work has been scientifically done, with great labor, much research and with careful comparison.

Operative dentistry has been systematized and its practice clarified. It has not been made easy, but positive and scientific.

The first essayist at the conclusion of his paper, said: "In closing, permit me to express my opinion on extension for prevention. It's success, if any, has been derived from the ninety and nine teeth that safely lay in the shelter of the fold—teeth that were never in danger; while the one lost sheep never entered the realm of consideration."

*Black, Page 142, Volume 2, Paragraph 2*:

"Extension for prevention is a definite requirement. The phrase as first used meant the laying of cavity margins at points, or along lines that would be seen by excursions of food. It has the definite object of preventing the recurrence of decay at the margins of fillings where recurrence of decay has been most often observed by so laying the margins as to obtain this cleaning effect. In proximal cavities the points were those which on account of near contact with proximating tooth surfaces, were not well cleaned by excursions of food in chewing, especially at the linguogingival, and labio- or bucco-gingival angles.—*A strict examination of ten thousand persons—discovered but nine persons* in whose teeth decay had spread superficially on the surface of the enamel across these angles."

We submit—does this look as if the lost sheep had received no consideration?

He further said: "To preserve all teeth, more especially a tooth that is about to be lost, should ever be our desire. To obtain such happy results, *excavate to preserve.*"

Our critic also says: "How satisfactory when working on frail teeth to excavate to preserve How unsafe to extend to prevent! The former method insures the best result possible, while the latter means the death of the tooth." But he gives no hint and refers to no authority for his phrase "Excavate to preserve," but let us see what relation Extension has to deep and dangerous cavities.

*Black, Page 225, Volume 1.*

"Extension for prevention has no relation whatever to the depth of the cavity. Every cavity prepared for a filling should be as shallow as the removal of all carious material will allow wherever this will give safe anchorage in dentin. This should be taken as an expression of a principle in all treatment of caries by filling. Large fillings extending far out into the buccal and labial surfaces should only be made when demanded by actual extension of decay."

Is not this a definition of excavation to preserve? Does it mean the death of the pulp or tooth as our writer puts it?

Our second essayest is a little more vigorous and explicit. He starts with a cavity for the purpose of illustrating—he says: "The reader may suppose he has a cavity on the approximal surface of a lower molar not larger than a pin head. Now in preparing this cavity the operator must bear in mind one thing—he is to reach immune surfaces, and he must not go below the thin shell of enamel. He starts first to prepare the main cavity and having settled this, he carries his drill through sound and healthy tissue until he reaches the supposed immune area—on the buccal and lingual surfaces. He does not know with any precision where said area is to be found, but supposing it may be on the convex surfaces of the tooth named or where the tongue or brush can have free access. Having completed the search for the so-called immunity on the surfaces named, the labor of discovery must be extended to the gingival border, for according to authority, immunity can alone be found under the gum and to insure this, the cavity must be carried below that tissue."

"Why this gingival cover should render a cavity immune is not explained. It is true it has been supposed to be answered repeatedly, and is again stated by Dr. Clack, who re-inforces his opinion by the quoted assertion of Dr. Black that these micro-organisms—those that produce acid —do not live under the free margin of the gum."

"Proceeding below the gingival border." "To proceed, to illustrate, the pin-head cavity is then carried down below the gingival border. To accomplish this, the said cavity must be extended to the dentin of the root, cutting away the cementum and pericementum, and dangerously encroaching on the pulp, and in any event subjecting that organ to thermal shocks that in due course of time will arouse inflammation there, and, ultimately, devitalization with pathological sequelae not pleasant to contemplate

In reply to this we would contend that there is no such a thing as a pin-head cavity on the proximal surface of any tooth—and to have made such an assertion is misleading. A good magnifying glass will reveal this fact to the satisfaction of any one who will take the trouble to make even a casual examination of a decay upon any smooth surface. When an excavator or explorer finds its way into the enamel, many others can also be found, and the entire unclean surface around, though the enamel rods may not have crumbled or be dislodged, is already involved in the carious process. Any system of cavity preparation which does not include the entire affected area, is worthless and unscientific.

*Black, Volume 1, Page 81.*

"If we examine the whitened outlines seen upon the surfaces (Proximal Surfaces) of molars and bicuspids of the teeth before any rods have fallen away, we will find these decays taking certain forms by reason of spreading, and often starting at several points instead of one central nidus."

*Black Volume 2, Page 123.*

"All cavities should be made as shallow as the removal of all decay will permit, provided good anchorage in dentin is obtained."

*Black, Volume 2, Page 201.*

"In the treatment of all gingival third and proximal cavities, it is an established fact that no decay begins, and that there is no recurrence of decay in any part of a tooth covered by a healthy free border of the gum."

*Black, Volume 2, Page 203.*

When "The examination of the overlapping gum tissues show that all of the gingival enamel has been destroyed and that the gingival walls of the cavity must be laid on the cementum—this is always a misfortune." Yet some critics would have us believe that the advocates of Extension teach that this is good practice.

Our second essayist further says: "The preparation of the pin head cavity is not however complete; the so-called immune surfaces have been reached and provided for, but the cavity over that surface of from one to two millimeters in depth, is not well fitted to receive a permanent filling—his experience leads him to conclude that it would be a dangerous experiment.

The pin head cavity is not complete for the occlusal surface with the fissures in the enamel is an ever present menace and must be removed. The filling and finishing of said filling is simply an effort of time and patience, but what is the condition of the pericementum left under the gingival border? No attention is paid to it.

The essayist further declares that this recital is not based on imagination, but is a description of a case in practice observed by him and carried out in minutest detail by the operator.

He further declares: "If our friends of the Extension—for—Prevention School object to it, they are bound to prove that their loose teaching through a catch phrase, does not lead to such fearful destruction of tooth tissue."

*Black, Volume 2, Page 143.*

"If this (Extension) can be had in narrow cavities, it is well. If it can be obtained by separating the teeth, and building prominent contacts, it is well. If the case requires wide cutting to accomplish it, that should be done. If the case is one that has become immune to caries, that fact may have consideration."

The writer's statement that his pin head example is a true report of an operation he witnessed, it is no intention of ours to deny, but that such an operation is in keeping with the teachings of the author, the quotations here presented, which were published in 1908, show the contrary most conclusively, and these might have been his if he had cared to possess them before giving to the world through the medium of the *Dental Cosmos* with its large circulation, such a misrepresentation of facts. To permit such statements to go unchallenged is to admit the truthfulness of them and leave many in doubt and ignorance as to the meaning and value of a principle of greatest importance in saving teeth, and at the same time discount the years of study and observation of one to whom we are greatly indebted.

To have written such misrepresentations when a knowledge of the true teachings were so easily to be had is dragging down and degrading a noble profession.

### DISCUSSION.

Dr. D. M. Cattell: I am very sorry by mistake I was not present and did not get to hear the paper. It is not my custom to not hear the paper, but as a Committeeman, I had other work to do. From the remarks I have gathered the paper was somewhat in defense of Dr. Black on his proposition in regard to extension for prevention, and I

am going to say now, the time has come when Dr. Black needs no defense. It is so well understood in the profession, so different from a few years ago, when he did need a defense. Why did he? Because men with the wrong ideas tried to get the profession to hear their teachings. They were only half informed themselves, so they created the wrong impression. There was a general discussion in New York and Philadelphia of what they were claiming to be Dr. Black's method of teaching extension for prevention. What was the result? It was a little debate. I wish I could repeat some of it but I can not now recall it. Thinking they had got the hang of Dr. Black's teachings they presumed to criticise it in their own way, but they mixed it up generally.

For several years they kept promulgating their wrong ideas. Finally through the influence of his friends Dr. Black was forced, I might say, to go to New York City and give them his teachings at first hand, as the old feelings still existed, which was a misunderstanding of the limitation of extension, that had resulted in a recognition of extension for prevention. Limitation was not referred to in the broad sense. I suppose that every critic of Black's teachings was a limited man that wanted to make clear his own particular ideas and he did not understand Black at all.

All who have followed the issues of it, or all who have read the literature of it, know well that the man who read the paper before the New York Dental Society discrediting Dr. Black's work discredits the best and most scientific work ever done for the profession on the subject.

Before he read that paper, there was nothing he had ever professed or done that gave him a right to pose as a critic of so scientific and conscientious a student as Dr. Black has always been.

Many men in Pennsylvania and the East, and Charles Tomes, of London, England, started in to prove Black's teachings false, but now they say we not only find that we were wrong, but we must praise everything that he has said. Nothing that has ever been asserted by Black has been disproved.

The majority of dentists have jumped at the conclusion that something is wrong with the extension principle, but I want to say that never yet has a single contradiction been made to the statements of principles that Dr. Black. has lain down, as based on scientific facts. No other man has done more for operative dentistry than Dr. Black And yet as a man who has understood the dentistry of this class and placed before the profession as Dr. Black. has the limitation of extension, shows that some men have gone wild on the proposition, not understanding it, yet so easy to understand, that no one should misconstrue his true meaning if he will take the trouble to read.

Dr. L. G. Noel: I did not have the pleasure of hearing all the paper, but I am in hearty defense of what I did hear, because we certainly all owe a great deal to that old man whose picture hangs in the corner.

I will say, whilst Dr. Black never expected us in all cases to fully carry out all the principles he so grandly planned, because we must put some common sense and some judgment in our work. He knows that it is impossible for us to work out those ideas he lays down in every case; still they are the highest ideals in dental practice. It would be very hard for us to work up to all his ideals, it, nevertheless, would be well for us to do so if we can. We all have nervous patients whose health is impaired by long operative cases, and it would not be well to attempt extensive excavations for these fillings are most liable to recurrence of decay.

I do not think that any one who understands Dr. Black can fail to credit the superiority of his teachings. I recognized the point in the paper that Dr. Morgan referred to; I got in while he was reading it, but I still say that we all owe our obligations to Dr. Black and are obliged to uphold his fine ideal teachings.

DR. W. C. GILLESPIE: I think Dr. Morgan's paper in defense of Dr. Black merits a good deal of consideration, and I would endorse any action we might take to uphold it, because Dr. Black's theory and teachings should be generally adopted.

In many instances extension for prevention is excessive as it involves excessive loss of tooth tissues. I think in many instances, it is necessary to pursue this outline for which reason, we naturally get better results. If our excavations are carried to the point outlined by Dr. Black there would be less decay than where it was left to each man's judgment without fixed rules for him to go by. Now, in teaching any theory, the teacher can not naturally afford to trample on the fundamental principles and the judgment of the author. He must lay down the theory clearly that applies to any operations.

All of Black's teaching is based upon sound general principles. I have read a good many articles by writers who usually discard Dr. Black's principles and they have done him a very great injustice, because of what he has given us as remarked in our general discussion. I thoroughly endorse Dr. Morgan's defense of it, and his remarks are severe enough to appear in any Dental Journal.

